# Editorial: The nocebo effect and its consequences for clinical trials and clinical practice

**DOI:** 10.3389/fpsyg.2022.1111324

**Published:** 2023-01-04

**Authors:** Karolina Wartolowska, Luana Colloca, Martina Amanzio

**Affiliations:** ^1^Nuffield Department of Clinical Neurosciences, University of Oxford, Oxford, United Kingdom; ^2^Department of Pain Translational Symptom Science and Placebo Beyond Opinions Center, University of Maryland School of Nursing, Baltimore, MA, United States; ^3^Department of Psychology, University of Turin, Turin, Italy; ^4^Neuroscience Institute of Turin (NIT), University of Turin, Turin, Italy

**Keywords:** nocebo, nocebo effects, negative placebo effects, side effects (SE), expectations

Recently, there has been an increase in interest in the nocebo effect, with a subsequent rise in the number of publications on the subject (Sweeney et al., 2022). Our recent Research Topic focuses on the nocebo effect in clinical trials and practice.

The concept of a nocebo effect is not new. It was first used in Kennedy (1961), who wrote “(…) it is somewhat surprising that little attention has been drawn to the existence of the contrary effect [to the placebo]—which I may call the nocebo reaction.” Kennedy recognized that the nocebo effect frequently contributes to the observed adverse effects but emphasized that these effects are inherent to the patient rather than the properties of the treatment and should not be confused with true pharmacological effects as this may lead to discarding useful drugs.

The nocebo effect is often called a negative placebo effect, but it is much more than just the flip side of the placebo effect. The nocebo effect causes negative or unfavorable reactions. These effects are not caused by the pharmacological or physical properties of a treatment, but they may resemble the effects of a treatment (Amanzio et al., 2009). Therefore, they are referred to as “non-specific side effects,” “adverse reactions of non-specific characters,” or “adverse non-drug reactions.” The nocebo effect sometimes leads to reduced treatment efficacy. Moreover, the nocebo effect is underpinned by different psycho-biological mechanisms than the placebo effect, further indicating that it is a separate phenomenon (Colloca and Barsky, 2020).

This Research Topic focuses on the nocebo effects in clinical trials and practice.

In a perspective review, Amanzio et al. described psychological distress from negative contextual factors during the pandemic COVID-19 as predisposing factors for the occurrence of the nocebo phenomenon. The media provided dramatic and negative descriptions that increased discomfort and anxiety and decreased response to treatment. Subsequently, data from randomized controlled trials of SARS-CoV-2 vaccines and from surveys of healthy individuals, health care workers, and patients with chronic pain disorders had confirmed this hypothesis (Amanzio et al., 2022).

A survey of university students and staff showed that a stronger belief of being infected with COVID-19, and potentially over-reporting of symptoms was linked to conscientiousness and health anxiety (Daniali and Flaten).

In their perspective article, Yetman et al. suggested that the nocebo effects evoked by information given by a healthcare professional may be affected by the perceived similarity or dissimilarity between the patient and the treatment provider, for example, different ethnicity. They called for more education of healthcare providers on the subject of nocebo, its links with clinical information about treatment and the potential strategies for management/mitigation.

The need for more extensive education on the subject of nocebo and its management was also emphasized in a survey of physiotherapists (Rossettini, Geri, et al.). The responders were aware of the existence of the nocebo effect, and only 18.6% said that it was “rarely,” and 1.4% that it was “never” present in their practice. They recognized the importance of the treatment provider and reported that they actively try to minimize the nocebo effect by managing patients' negative expectations.

A series of experiments by Zech, Scharl, et al., Zech, Schrödinger, Hansen, and Zech, Schrödinger, Seemann, et al. demonstrated that negative information increase anxiety but also have a detrimental effect on functional measures such as muscle strength. People with higher health anxiety, tend to report more negative symptoms and this effect persists even after controlling for generalized anxiety and depression and independently of the potential for a financial reward through litigation (Lecci et al.). Anxiety and fear learning after verbal suggestion are stronger in delusion-prone people (Louzolo et al.).

The effect between the verbal suggestions and the reported negative symptoms, e.g., itch, is mediated by expectations (Meeuwis et al.). Once generated, treatment-related expectations are difficult to modify and may persist—even when proven not to be supported by evidence (Rossettini, Colombi, et al.).

A study in patients under general anesthesia undergoing a surgical procedure has shown that verbal suggestions given to sedated patients may reduce post-operative nausea and vomiting (Nowak et al.).

There are two very positive aspects of this Research Topic that are worth highlighting. Firstly, the included articles demonstrated the ubiquitous and heterogeneous nature of nocebo—not just as a negative response to a placebo but also as adverse effects of treatment and common symptoms misattributed to treatment or disease. For example, the publications were concerned with the nocebo effects in various contexts: from experimental studies with an inert placebo, through side effects of treatment, to COVID-19 symptoms. These studies investigated the associations between nocebo effects and suggestions, expectations, health anxiety, personality factors, and racial/ethnic differences. Secondly, unlike most of the existing literature on the subject, which is dominated by reviews and opinion papers, most of the included studies were primary data-based articles. For example, two-thirds of the articles reported the results of experimental studies, including one, which used neuroimaging to explain further the mechanisms linking fear learning with the nocebo effect. There were also two surveys, one of the healthcare providers and one of the public. There is an urgent need for more good quality mechanistic research studies designed to investigate factors responsible for nocebo effects.

However, this Research Topic also reflects some of the problems with the existing research on the nocebo effects. Firstly, many of the experimental studies reported *post-hoc* and secondary analyses of studies rather than primary analyses—emphasizing the lack of experimental studies in clinical populations specifically designed to investigate the nocebo effect as the primary outcome rather than as an afterthought. Secondly, many purposefully designed studies were often in healthy controls and attempted to generalize findings from healthy controls to clinical populations. Finally, there is a need for a standardized definition of the nocebo effect. Defining the nocebo effect in the context of placebo obscures the fact that it is a separate problem with far more serious consequences for clinical practice and research. Furthermore, referring to the same phenomenon by many different names hinders the development of a standard definition of nocebo and a consolidated analysis of the research on the subject.

In summary, this Research Topic has demonstrated an increasing recognition of the complex nature of nocebo and the current gaps in both clinical practice and research.

## Author contributions

KW drafted the manuscript. KW, MA, and LC edited the manuscript and revised it for important intellectual content. All authors contributed to the article and approved the submitted version.
